# Human NANOS1 Represses Apoptosis by Downregulating Pro-Apoptotic Genes in the Male Germ Cell Line

**DOI:** 10.3390/ijms21083009

**Published:** 2020-04-24

**Authors:** Damian M. Janecki, Erkut Ilaslan, Maciej J. Smialek, Marcin P. Sajek, Maciej Kotecki, Barbara Ginter-Matuszewska, Patryk Krainski, Jadwiga Jaruzelska, Kamila Kusz-Zamelczyk

**Affiliations:** 1 Institute of Human Genetics, Polish Academy of Sciences, Strzeszynska 32, 60-479 Poznan, Poland; erkut.ilaslan@igcz.poznan.pl (E.I.); maciej.smialek@igcz.poznan.pl (M.J.S.); marcin.sajek@igcz.poznan.pl (M.P.S.); jadwiga.jaruzelska@igcz.poznan.pl (J.J.); 2 Institute of Bioorganic Chemistry, Polish Academy of Sciences, Noskowskiego 12/14, 61-704 Poznan, Poland; 3 Department of Developmental, Molecular and Chemical Biology, Tufts University Medical School, 136 Harrison Ave, Boston, MA 02111, USA; mkkotecki@gmail.com; 4 Department of Histology and Embryology, Poznan University of Medical Sciences, Swiecickiego 6, 60-781 Poznan, Poland; bgintermatuszewska@ump.edu.pl; 5 Department of Clinical Pathology, Poznan University of Medical Sciences, Przybyszewskiego 49, 60-355 Poznan, Poland; krainski.patryk@gmail.com

**Keywords:** NANOS1, apoptosis, cell cycle, human infertility, NANOS1 mutation

## Abstract

While two mouse NANOS paralogues, NANOS2 and NANOS3, are crucial for maintenance of germ cells by suppression of apoptosis, the mouse NANOS1 paralogue does not seem to regulate these processes. Previously, we described a human NANOS1 p.[(Pro34Thr);(Ser83del)] mutation associated with the absence of germ cells in seminiferous tubules of infertile patients, which might suggest an anti-apoptotic role of human NANOS1. In this study, we aimed to determine a potential influence of human NANOS1 on the maintenance of TCam-2 model germ cells by investigating proliferation, cell cycle, and apoptosis. Constructs encoding wild-type or mutated human NANOS1 were used for transfection of TCam-2 cells, in order to investigate the effect of NANOS1 on cell proliferation, which was studied using a colorimetric assay, as well as apoptosis and the cell cycle, which were measured by flow cytometry. RNA-Seq (RNA sequencing) analysis followed by RT-qPCR (reverse transcription and quantitative polymerase chain reaction) was conducted for identifying pro-apoptotic genes repressed by NANOS1. Here, we show that overexpression of NANOS1 downregulates apoptosis in TCam-2 cells. Moreover, we found that NANOS1 represses a set of pro-apoptotic genes at the mRNA level. We also found that the infertility-associated p.[(Pro34Thr);(Ser83del)] mutation causes NANOS1 to functionally switch from being anti-apoptotic to pro-apoptotic in the human male germ cell line. Thus, this report is the first to show an anti-apoptotic role of NANOS1 exerted by negative regulation of mRNAs of pro-apoptotic genes.

## 1. Introduction

Apoptosis is an essential process in male germ cell development. This kind of cell death controls the correctness and number of germ cells and occurs during both the migration of primordial germ cells (PGCs) to primary gonads and spermatogenesis. The final number of germ cells in the testes is determined by a tightly regulated balance between cell proliferation and apoptosis. Consequently as many as 75% of germ cells are eliminated by apoptosis in healthy vertebrate testes. Imbalance between proliferation and apoptosis can lead to a decreased number of germ cells manifested clinically as infertility, while an uncontrolled increase in the number of these cells may lead to testicular germ cell tumors (TGCT); for a review, see [1]. Thus, expanding the knowledge of molecular factors influencing germ cell apoptosis is crucial for the development of novel therapeutic strategies for both male infertility and TGCT.

Nanos proteins are widespread within the animal kingdom, and their critical function for apoptosis regulation in germ cells, e.g., an anti-apoptotic role in *Drosophila* germ cells, is well established. Nanos proteins act as post-transcriptional repressors of specific mRNAs by binding to them using highly conserved zinc-finger domain; for a review, see [2]. In particular, *Drosophila* Nanos was shown to repress caspase activators such as *hid* (*head involution defective*) and *skl* (*sickle*) to inhibit apoptosis in PGCs. This inhibition is crucial for the survival of PGCs during migration to the primary gonads [3]. Nanos knockout in *Drosophila* leads to infertility in both sexes caused by the lack of germ cells [4]. An anti-apoptotic property of Nanos is conserved in evolution at least from *Drosophila* to mice. There are three NANOS paralogues in mammals and two of them, NANOS2 and NANOS3, play anti-apoptotic roles in different stages of germ cell lineage development in mice [5,6,7]. Apoptosis of PGCs is suppressed by NANOS3 [5], and knockout of the murine gene causes infertility in both sexes [6]. In turn, NANOS2 represses apoptosis, specifically of male gonocytes, and its knockout in mice causes infertility restricted to male sex [6,7]. By contrast to *Drosophila,* to the best of our knowledge, specific mRNAs encoding apoptotic factors controlled by NANOS2 or NANOS3 in mammals have not been revealed. Unlike *Nanos2* and *Nanos3* knockout resulting in mice infertility, *Nanos1* knockout mice are viable and fertile, indicating that the NANOS1 protein is dispensable for mouse development and fertility [8].

While NANOS proteins have also been implicated in human germ cell development [9,10,11,12], their functions identified in other species, such as regulation of apoptosis, have hardly been characterized in humans. So far, only the expression profile of human *NANOS* paralogues and the association between *NANOS* mutations and infertility have been investigated. Similar to the mouse orthologue, human *NANOS2* was shown to be expressed specifically in male germ cells, indicating a potential association between *NANOS2* mutations and male infertility. However, the detected mutations in infertile patients seem not to be causative [12]. In turn, human *NANOS3* expression was shown in fetal and adult gonads as well as in the adult brain [10,11]. *NANOS3* gene mutations were found in a group of infertile men, but no causation was detected [11]. On the other hand, two out of four *NANOS3* mutations were detected to be linked to premature ovarian insufficiency (POI) in infertile women [13,14,15,16]. Moreover, Santos et al. demonstrated that one *NANOS3* mutation linked to POI causes increased apoptosis of cultured cells, suggesting an anti-apoptotic role of human NANOS3 [14], as was shown for a mouse orthologue.

**Figure 1:**
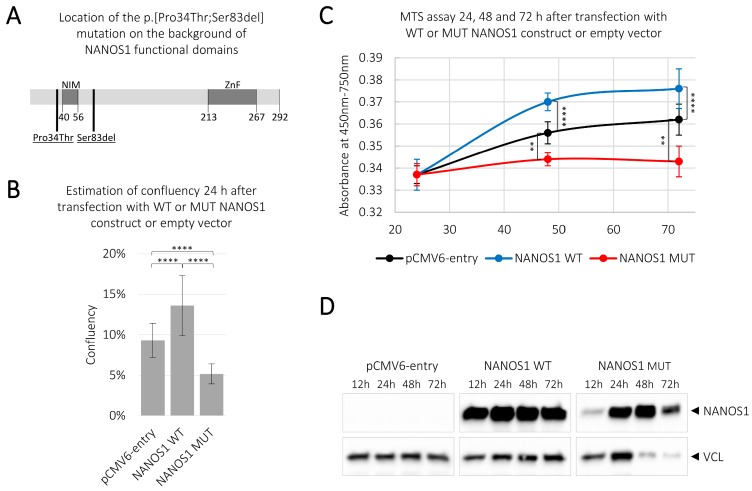
Influence of the wild-type and the mutated NANOS1 p.[(Pro34Thr);(Ser83del)] on cell confluency and proliferation. TCam-2 cells were transfected with plasmids encoding the wild-type (WT) NANOS1, the mutated (MUT) NANOS1 or the empty pCMV6-entry vector. (**A**) Scheme of the NANOS1 protein. NOT1 interacting motif (NIM) domain, zinc-finger (ZnF) domain, and amino acid position of the double mutation p.[(Pro34Thr);(Ser83del)] are indicated. (**B**) Confluency measurement 24 h after transfection using JuLI™ FL Fluorescence Cell History Recorder. (**C**) The MTS assay was used to analyze cell proliferation of TCam-2 cells 24, 48, and 72 h after transfection. A similar number of cells counted 12 h after transfection was used for normalization of each experiment. This experiment was performed in three biological replicates. (**D**) The expression of the wild-type and the mutated NANOS1 constructs after transfection compared to the negative control was analyzed by western blot analysis using an anti-DDK antibody. As a loading control, VCL (vinculin) protein was used, in each sample shown in the lower panel. A *p* value < 0.05 was considered statistically significant, a *p* value < 0.005 was marked by two asterisks (**), and a *p* value < 0.00005 by four asterisks (****).

Although the mouse *Nanos1* orthologue seems not to be critical for germ cell development and human *NANOS1* expression is more ubiquitous than *NANOS2* and *NANOS3* [10], one out of *NANOS1* mutations was found to be potentially causative among a group of infertile men. Namely, a NM_199461.4(NANOS1_v001):c.[100C>A;240_242del];NM_199461.4(NANOS1_i001):p.[(Pro34Thr);(Ser83del)] double mutation (in this report referred to as p.[(Pro34Thr);(Ser83del)]) was identified in two infertile male patients manifesting in the absence of germ cells in semen and seminiferous tubules (Sertoli cell only syndrome–SCOS) [17]. This mutation encompasses the N-terminal conserved NIM (NOT1 interacting motif) region (Figure 1A), which is necessary for recruitment of the deadenylase complex to deadenylate, and leads to degradation of mRNA targets [18]. Interestingly, the p.[(Pro34Thr);(Ser83del)] double mutation is located in a NANOS1 region which is not present in the mouse orthologue [17]. The difference in the structures of the mouse and human NANOS1 protein could reflect a distinct significance of these NANOS1 orthologues for germ cell development. The SCOS associated with the *NANOS1* mutation suggests the critical role of this gene in maintenance of the germline in men, but specific biological processes controlled by NANOS1 have remained elusive.

The aim of this work was to determine the potential influence, and its mechanism, of human NANOS1 on maintenance of germ cells. We show that the NANOS1 protein suppresses apoptosis of germ cells in culture through repression, at the mRNA level, of a group of pro-apoptotic genes. Moreover, we demonstrate that the aforementioned p.[(Pro34Thr);(Ser83del)] mutation reverses NANOS1 activity from anti-apoptotic to pro-apoptotic by disrupting NANOS1 repression of some pro-apoptotic genes.

## 2. Results

### 2.1. Overexpression of the Wild-Type NANOS1 Increases, While the Mutated NANOS1 p.[(Pro34Thr);(Ser83del)] Decreases, the Proliferation of TCam-2 cells

In order to examine the potential influence of NANOS1 on the viability of germ cells as well as the functional significance of the NANOS1 double mutation (Figure 1A), we studied the wild-type and the mutated NANOS1 effects on the proliferation status of the TCam-2 cell line. This cell line originates from human seminoma and represents male germ cells at an early stage of development as PGCs/gonocytes [19]. Our transcriptome analysis revealed that NANOS1 expression is very low at the mRNA level (1.76 FPKM–fragments per kilobase of transcript per million mapped reads) in TCam-2 cells [20]. For that reason, instead of using a knock-down strategy, we decided to overexpress the wild-type and the mutated NANOS1 in TCam-2 cells in order to markedly alter the expression level and to investigate the function of this protein in human germ cells. While overexpression of the wild-type NANOS1 caused a significant increase (of nearly 50%) in cell confluency compared to the empty vector, overexpression of the mutated NANOS1 caused a significant decrease (of nearly 45%) in the cell confluency, as compared to the empty vector (Figure 1B). To check whether the above effects on the cell confluency reflect the influence of NANOS1 on cell proliferation, we overexpressed the wild-type and the mutated NANOS1 in TCam-2 cells and performed a colorimetric assay (MTS assay). We found that the wild-type NANOS1 stimulated cell proliferation, whereas the mutated NANOS1 caused a significant decrease in cell proliferation, as compared to the empty vector (Figure 1C). Both the cell confluency and the MTS assay consistently showed that NANOS1 may maintain germ cells by pro-proliferative activity, whereas its mutation reverts this ability. Expression of both the NANOS1 constructs after transfection is shown in Figure 1D.

### 2.2. Overexpression of Both the Wild-Type and the Mutated NANOS1 Inhibits the Cell Cycle of TCam-2 cells

**Figure 2:**
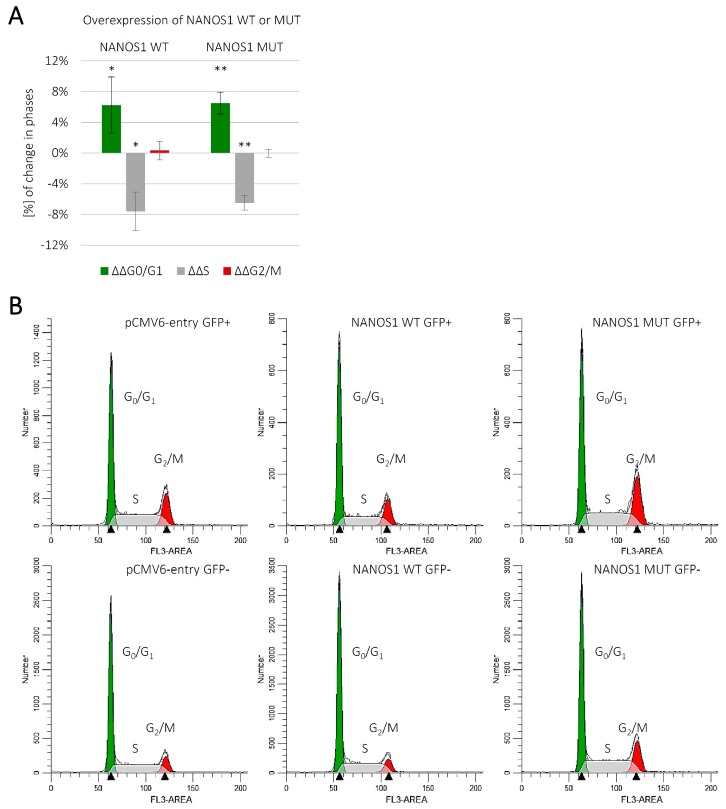
Cell cycle analysis of propidium iodide-stained TCam-2 cells by flow cytometry. (**A**) The graph represents distribution at G0/G1, S, and G2/M cell cycle phases of unsynchronized TCam-2 cells overexpressing the wild-type (WT) NANOS1 or the mutated (MUT) NANOS1 p.[(Pro34Thr);(Ser83del)] compared to the empty pCMV6-entry control vector (baseline). Flow cytometry measurement of the distribution was performed 48 h after transfection with the construct encoding the wild-type, the mutated NANOS1 or the empty pCMV6-entry vector co-transfected with GFP-F. Percentages of cells in each cell cycle phase were calculated using ModFit LT 5.0 software. This experiment was repeated in four independent replicates. A *p* value < 0.05 was considered statistically significant and was marked by one asterisk (*), and a *p* value < 0.005 was marked by two asterisks (**). (**B**) Histogram representation of the TCam-2 cell distribution at G0/G1, S, and G2/M phases of the cell cycle in unsynchronized culture upon transfection with the construct encoding the wild-type (WT) or the mutated (MUT) NANOS1 or the empty pCMV6-entry vector co-transfected with GFP-F. Histograms were generated by ModFit LT 5.0 software and show one representative example from four independent experiments.

Since we observed increased proliferation upon overexpression of the wild-type NANOS1 (Figure 1C), we addressed the question whether that effect was due to modulation of the cell cycle and, if so, whether the p.[(Pro34Thr);(Ser83del)] mutation affected such a modulation. We performed flow cytometric analysis of the cell cycle distribution in unsynchronized TCam-2 cells overexpressing the wild-type or the mutated NANOS1, in comparison with the empty vector (Figure 2A). The separation of cell populations in each cell cycle phase was performed using ModFit LT 5.0 software (Figure 2B). Upon overexpression of the wild-type NANOS1, we observed an increase in the proportion of cells in the G0/G1 phase, with a concordant decrease in the proportion of cells in the S phase, as compared to the empty vector (Figure 2A), indicating that NANOS1 inhibits the TCam-2 cell cycle. We show, for the first time, the effect of any Nanos protein on cell cycle distribution. However, the inhibition of the cell cycle by NANOS1 does not explain its pro-proliferative role. Moreover, no significant difference in cell cycle effects was observed between cells expressing the wild-type and the mutated NANOS1 (Figure 2A).

### 2.3. Overexpression of the Wild-Type NANOS1 Decreases, Whereas the Mutated NANOS1 p.[(Pro34Thr);(Ser83del)] Increases Apoptosis of TCam-2 cells

**Figure 3:**
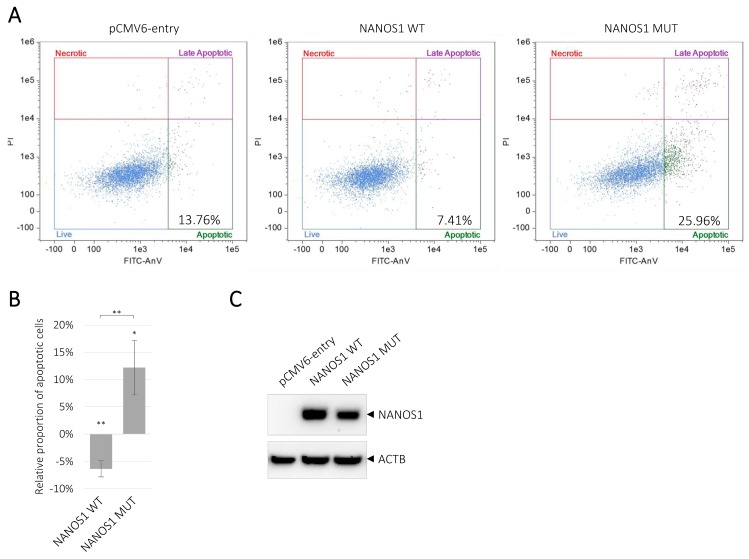
Influence of the wild-type and the mutated NANOS1 p.[(Pro34Thr);(Ser83del)] on apoptosis of TCam-2 cells. The apoptosis was measured by using Annexin V staining and flow cytometry. (**A**) The representation of dot plots generated by flow cytometric analysis of the apoptosis in TCam-2 cells upon transfection of the wild-type (WT), the mutated (MUT) NANOS1 or the empty pCMV6-entry plasmid. The percentages of apoptotic cells are shown in each plot. (**B**) The dot plots data quantitation that represents the proportion of apoptotic cells in TCam-2 cells transfected with the wild-type or the mutated NANOS1, relative to cells transfected with the empty vector (baseline). (**C**) Western blot analysis after transfection of TCam-2 cells with the pCMV6-entry vector, the wild-type or the mutated NANOS1. As a loading control, we used ACTB (actin beta) protein, in each sample shown in the lower panel. These experiments were performed in three biological replicates. A *p* value < 0.05 was considered statistically significant and was marked by one asterisk (*), and a *p* value < 0.005 was marked by two asterisks (**).

We investigated the function of NANOS1 in the maintenance of germ cells by studying the NANOS1 effect on apoptosis. To determine whether the divergent effects of the wild-type and the mutated NANOS1 on cell proliferation (Figure 1C) were associated with a different influence on apoptosis, we performed flow cytometry analysis of Annexin V-stained TCam-2 cells transfected with the wild-type or the mutated NANOS1, as compared to the empty vector. We showed that the wild-type NANOS1 reduced the apoptosis of TCam-2 cells. To the best of our knowledge, this is the first evidence for any human NANOS to suppress apoptosis. Moreover, we found that the mutated NANOS1 induced apoptosis (Figure 3A). Thus, we showed that the NANOS1 anti-apoptotic activity was reversed to pro-apoptotic activity by the mutation. A quantification of the flow cytometric data is presented on the graph in Figure 3B, while the expression of the wild-type and the mutated NANOS1 proteins in transfected cells is shown in Figure 3C.

### 2.4. NANOS1 Negatively Regulates Pro-Apoptotic Genes and This Regulation is Disrupted by the p.[(Pro34Thr);(Ser83del)] Mutation

**Figure 4:**
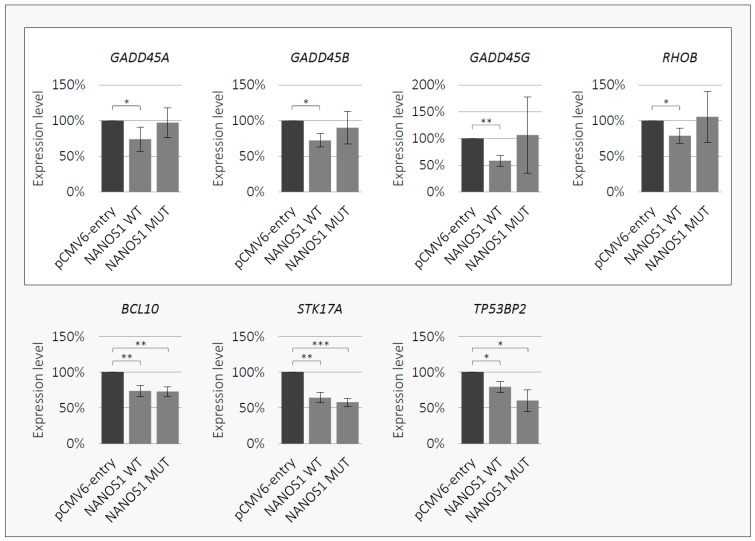
RT-qPCR analysis of selected pro-apoptotic genes in TCam-2 cells overexpressing the empty pCMV6-entry vector, the wild-type (WT) or the mutated (MUT) NANOS1 p.[(Pro34Thr);(Ser83del)]. Repression of seven mRNAs by the wild-type NANOS1 is shown in the grey box, while disruption of the repression of four mRNAs by the mutated NANOS1 is shown in the white box. A *p* value < 0.05 was considered statistically significant and was marked by one asterisk (*), a *p* value < 0.005 was marked by two asterisks (**), and a *p* value < 0.0005 by three asterisks (***).

Given that the NANOS1 protein is a posttranscriptional regulator of gene expression [18], we performed a search for apoptosis-related transcripts regulated by NANOS1 in order to elucidate its anti-apoptotic property. Therefore, we carried out RNA-Seq analysis of the wild-type NANOS1 overexpression in TCam-2 cells (RNA-Seq data are available from the Gene Expression Omnibus, accession GSE134802). In this way, we identified 10 mRNAs encoding pro-apoptotic factors (*GADD45A*, *GADD45B*, *GADD45G*, *RHOB*, *BCL10*, *STK17A*, *TP53BP2*, *RIPK1*, *SIAH1*, *JUN*), which were downregulated upon NANOS1 overexpression (Table 1). We confirmed downregulation of seven of them (*GADD45A*, *GADD45B*, *GADD45G*, *RHOB*, *BCL10*, *STK17A*, *TP53BP2*) using RT-qPCR (Figure 4). Thus, for the first time, we identified genes encoding pro-apoptotic factors to be downregulated by NANOS1. To the best of our knowledge, this is also the first identification of any apoptosis-related mRNAs regulated by any Nanos protein among vertebrates. Finally, we investigated whether the p.[(Pro34Thr);(Ser83del)] mutation affects NANOS1-mediated regulation by performing RT-qPCR upon overexpression of the mutated NANOS1. We found that the regulation of four mRNAs (*GADD45A*, *GADD45B*, *GADD45G*, *RHOB*) was abrogated by the p.[(Pro34Thr);(Ser83del)] NANOS1 mutation (Figure 4, upper panel). Interestingly, all of these mRNAs encode proteins of closely related functions, namely, factors activating DNA damage-induced apoptosis.

## 3. Discussion

Regulation of germ cell number is critical for both fertility and prevention of TGCT. Some Nanos proteins are known to play a critical role in this regulation by suppression of germ cell apoptosis. In the mouse model, NANOS2 and NANOS3 paralogues were shown as apoptosis suppressors, but the molecular background of this activity has not been explained [5,6,7]. In turn, mouse NANOS1 seems to be unnecessary for regulation of germ cell number since *Nanos1* knockout animals were fertile and healthy [8]. Recently, we described a *NANOS1* gene p.[(Pro34Thr);(Ser83del)] mutation associated with the lack of germ cells in seminiferous tubules in infertile SCOS patients, suggesting a possible anti-apoptotic role of the human NANOS1 homologue [17].

Here, we investigated the potential influence of human NANOS1 on the maintenance of germ cells showing that NANOS1 overexpression increases the number of viable male germ cells in culture. In order to shed light on this phenomenon, we studied the NANOS1 influence on the cell cycle and apoptosis. We showed that overexpression of NANOS1 caused a slight increase in the proportion of cells in the G0/G1 phase, with a concordant decrease of cells in the S phase. This is the first report, to the best of our knowledge, demonstrating quantification of cells in various phases of the cell cycle in response to NANOS proteins. Our result indicates that slight NANOS1-mediated cell cycle inhibition is accompanied by stronger NANOS1 inhibition of apoptosis, which accounts for the overall NANOS1 positive role in cell proliferation. It is the first report showing an anti-apoptotic and pro-proliferative role of any human NANOS paralogue and, more broadly, any NANOS1 mammalian orthologue. We hypothesized that NANOS1 negatively regulates apoptosis by repressing mRNAs encoding pro-apoptotic factors. Indeed, we identified seven mRNAs encoding well-established pro-apoptotic factors to be downregulated by NANOS1: *GADD45A*, *GADD45B*, *GADD45G*, *RHOB*, *BCL10*, *STK17A,* and *TP53BP2*. Downregulation of these mRNAs by NANOS1 may account for its strong anti-apoptotic properties in human male germ cells. Regulation of mRNAs encoding apoptosis-promoting factors by any mammalian NANOS homologue has never been reported. Although we observed downregulation of the above-mentioned pro-apoptotic mRNAs caused by NANOS1 overexpression, we did not explore whether NANOS1 regulates them directly or indirectly. Therefore, it requires further investigation to establish whether these mRNAs are direct targets of NANOS1. Importantly, we observed that the *NANOS1* p.[(Pro34Thr);(Ser83del)] mutation increased apoptosis of human male germline cells in culture. This suggests that this mutation provoked a functional reversal of NANOS1 from anti-apoptotic to pro-apoptotic. We also found that this mutation disrupted NANOS1 inhibition of four pro-apoptotic mRNAs, namely, *GADD45A*, *GADD45B*, *GADD45G,* and *RHOB*. These genes seem to be the most specifically responsible for the NANOS1 anti-apoptotic function. Interestingly, each of these four mRNAs encodes a protein activating DNA damage-induced apoptosis. Their transcription is upregulated in response to genotoxic stress [21,22,23]. The three GADD45 (growth arrest and DNA damage inducible 45) protein family members induce apoptosis by binding and activating MAP3K4 kinase, which in turn positively regulates the p38/JNK-mediated apoptotic pathway [21,24]. RHOB (ras homologue family member B), in turn, activates apoptosis by positive regulation of JNK that triggers the activation of pro-apoptotic BCL2L11 [25,26]. We propose that abrogated inhibition of the above-mentioned four pro-apoptotic mRNAs upon expression of mutated NANOS1 accounts for increased apoptosis of TCam-2 cells. The functional switch of NANOS1 from anti-apoptotic to pro-apoptotic may represent the mechanism underlying the infertility in patients with the *NANOS1* p.[(Pro34Thr);(Ser83del)] mutation. In patients, this mutation could affect the regulation of *GADD45A*, *GADD45B*, *GADD45G,* and *RHOB* at the post-transcriptional level, resulting in upregulated expression of encoded pro-apoptotic factors, thereby causing increased apoptosis of germ cells and finally, the absence of germ cells from seminiferous tubules and infertility.

It is important to note that the human male germ cell line TCam-2 originates from seminoma, which is a type of TGCT [19]. In this context, it is worth noting that almost all NANOS1-repressed pro-apoptotic factors identified in this study (GADD45A, GADD45B, GADD45G, RHOB, and TP53BP2) are well-established tumor suppressors [27,28,29,30,31,32,33,34,35]. This is in line with NANOS1 itself being proposed to promote carcinogenesis [36]. Therefore, it is likely that in addition to playing a role in germ cell development, NANOS1 downregulates the expression of tumor-suppressing factors, as has already been suggested for other RNA-binding proteins [37]. This finding represents an interesting issue for future studies aimed at investigating the role of NANOS1 in carcinogenesis.

## 4. Materials and Methods

### 4.1. Cell Culture and Transfection

TCam-2 cells were obtained from Dr. Sohei Kitazawa and were maintained in RPMI with GlutaMAX medium (Gibco, Life Technologies, 61870044, Paisley, UK) supplemented with 10% (*v*/*v*) foetal bovine serum (GE Healthcare HyClone, SH30071, Logan, Utah, USA) and 1% (*v*/*v*) antibiotic antimycotic solution (Lonza, EE17-602E, Germany). The cells were transfected with plasmid constructs using the Neon Transfection System (Thermo Fisher Scientific, Waltham, Massachusetts, USA) according to the manufacturer’s protocol.

### 4.2. Estimation of Cell Confluency

TCam-2 cells (2 × 10^5^) were transfected with 1.5 µg of constructs encoding the wild-type, the mutated NANOS1 p.[(Pro34Thr);(Ser83del)] or the empty pCMV6-entry vector (OriGene Technologies, PS100001, Rockville, Maryland, USA) in three biological replicates (three independent transfections). Cell confluency was measured 24 h after transfection in five random areas of each 6-well plate using the JuLI™ FL Fluorescence Cell History Recorder (NanoEnTek, Seoul, South Korea).

### 4.3. MTS Assay

TCam-2 cells (2 × 10^5^) were transfected with 1.5 µg of constructs encoding the wild-type, the mutated NANOS1 p.[(Pro34Thr);(Ser83del)] or the empty pCMV6-entry vector (OriGene Technologies, PS100001, Rockville, Maryland, USA) in three biological replicates (three independent transfections; five technical repeats in each of five different wells of a 96-well plate) and were cultured for 24, 48, and 72 h after transfection. In order to obtain a similar number per well of viable cells expressing the different constructs for the MTS assay, viable cells were counted 12 h post-transfection using Trypan Blue staining. Similar numbers of cells were seeded in five different wells of a 96-well plate. The cell viability was measured using GloMax®-Multi Detection System (Promega, Madison, Wisconsin, USA) at a 450 nm (and 750 nm for the cell background) wavelength 24, 48, and 72 h after transfection and 4 h after adding 20 µl of CellTiter 96^®^ AQ One Solution Reagent (Promega, Madison, Wisconsin, USA) to each well containing transfected TCam-2 cells. The overexpression of the wild-type and the mutated NANOS1 was measured 12, 24, 48, and 72 h after transfection by western blot using an anti-DDK antibody (OriGene Technologies, TA50011, Rockville, Maryland, USA). VCL (vinculin) protein was used as a loading control.

### 4.4. Western Blotting

Overexpression efficiency was measured by western blot analysis under standard conditions, using a nitrocellulose membrane and horseradish peroxidase (HRP)-conjugated secondary antibodies. Semi-quantitative measurement of protein levels was performed using ImageLab 5.1 software (Bio-Rad, Hercules, California, USA). The chemiluminescent signal was detected using the Clarity™ Western ECL Substrate for HRP (Bio-Rad, 1705060, Hercules, California, USA).

### 4.5. Antibodies

The primary antibody anti-DDK (FLAG^®^) (OriGene Technologies, TA50011, 1:2500, Rockville, Maryland, USA) was used for detection of proteins expressed with the pCMV6-entry vector. Anti-ACTB (actin beta) (Sigma-Aldrich, A2066, 1:10000, Saint Louis, Missouri, USA), and anti-VCL (vinculin) (Abcam, ab129002, 1:20000, Cambridge, UK) antibodies were used as loading controls. Secondary antibodies, i.e., goat anti-rabbit IgG-HRP (Sigma-Aldrich, A6154, 1:250000, Saint Louis, Missouri, USA) and goat anti-mouse IgG-HRP (Santa Cruz Biotechnology, sc-2005, 1:10000, Dallas, Texas, USA), were used.

### 4.6. Flow Cytometry

Detection of apoptotic TCam-2 cells was performed 48 h after transfection of 3 × 10^5^ TCam-2 cells with 2 µg of constructs encoding the wild-type, the mutated NANOS1 p.[(Pro34Thr);(Ser83del)] or the empty pCMV6-entry vector (OriGene Technologies, PS100001, Rockville, Maryland, USA). Cells were stained using the Annexin V-FITC Apoptosis Detection Kit (Beckman Coulter, Brea, California, USA) according to the manufacturer’s protocol and were measured by flow cytometry using a FlowSight instrument (Amnis, Seattle, Washington, USA). The results were analyzed using Image Data Exploration and Analysis Software version 6.0 (IDEAS^®^ v6.0, Amnis, Seattle, Washington, USA). The experiment was performed in three biological replicates. Cell cycle analysis was performed 48 h after transfection of 2 × 10^6^ TCam-2 cells with 20 µg of constructs encoding the wild-type, the mutated NANOS1 p.[(Pro34Thr);(Ser83del)] or the empty pCMV6-entry vector co-transfected with GFP-F at a proportion of 9:1. For this purpose, TCam-2 cells were washed with PBS and fixed in a cold 100% methanol on ice for 10 min. After incubation at 37 °C for 15 min in 50 µg/mL propidium iodide (PI) (Sigma-Aldrich, P4170, Saint Louis, Missouri, USA) containing 330 µg/mL RNase A (Sigma-Aldrich, R6513, Saint Louis, Missouri, USA), cells were incubated for 1 h on ice and finally measured using the S3e™ Cell Sorter (Bio-Rad, Hercules, California, USA) apparatus. Data files were analyzed using ModFit LT™ 5.0 (Verity Software House, Topsham, Maine, USA). The experiment was performed in four biological replicates.

### 4.7. cDNA Library Preparation and RNA-Seq

TCam-2 cells (2 × 10^5^) were transfected with 1.5 µg of constructs encoding the wild-type NANOS1 or the empty pCMV6-entry vector in three biological replicates. Transcription was stopped by Actinomycin D (5 µg/mL)(Sigma-Aldrich, A1410, Saint Louis, Missouri, USA) 24 h after transfection for 4 h. Total RNA was extracted using an RNeasy Plus Micro Kit (Qiagen, Hilden, Germany) according to the manufacturer’s protocol. RNA quality was checked on a Bioanalyzer (Agilent Technologies, Santa Clara, California, USA) using the RNA 6000 Nano Kit (Agilent Technologies, Santa Clara, California, USA). Total RNA samples with RIN value > 7 were used for library preparation. cDNA libraries were prepared using the TruSeq RNA Sample Prep v2 (Illumina, San Diego, Santa Clara, California, USA), and subsequent next-generation sequencing (PE:100 nt) was performed on an Illumina HiSeq 4000 platform by Macrogen (Seoul, South Korea). RNA-Seq data described in this study are available from the Gene Expression Omnibus (accession GSE134802).

### 4.8. Bioinformatic Analysis

Differential expression analysis was performed on the Galaxy platform (Pennsylvania State University, State College, Pennsylvania, USA; Johns Hopkins University, Baltimore, Maryland, USA; Oregon Health and Science University, Portland, Oregon, USA) by the following pipeline: Paired-End sequences obtained from the HiSeq 4000 platform were trimmed using Trimmomatic v0.36.3 [38]. Sequence reads that passed quality filters were mapped to the human reference genome hg38 using Bowtie 2 v2.3.4.1 [39]. Mapped reads from three biological repetitions of NANOS1 and empty vector overexpression libraries were counted using featureCounts v1.6.0.3 [40] followed by calculation of the differential gene expression with DESeq2 v2.11.40.1 [41]. Differentially expressed transcripts were filtered according to the parameters log2FC ≤ −0.3 and adjusted *p* value ≤ 0.05.

### 4.9. Quantitative RT-PCR

TCam-2 cells (2 × 10^5^) were transfected with 1.5 µg of constructs encoding the wild-type, the mutated NANOS1 p.[(Pro34Thr);(Ser83del)] or the empty pCMV6-entry vector in three biological replicates. Transcription was stopped by Actinomycin D (5 µg/mL) (Sigma-Aldrich, A1410, Saint Louis, Missouri, USA) 24 h after transfection for 4 h. Total RNA isolation, template preparation, and RT-qPCR reactions conditions were described previously [42]. Sequences of the primers are listed in Supplementary Table S1. ARNT, GAPDH, RPL13, and UBC genes were used for normalization.

### 4.10. Constructs

For the wild-type and the mutated NANOS1 p.[(Pro34Thr);(Ser83del)] overexpression, we used constructs previously described [43].

### 4.11. Accession Numbers

NANOS1 NM_199461.4, GADD45A NM_001924.4, GADD45B NM_015675.4, GADD45G NM_006705.4, RHOB NM_004040.4, BCL10 NM_003921.5, STK17A NM_004760.3, TP53BP2 NM_001031685.3, RIPK1 NM_003804.6, SIAH1 NM_003031.4, JUN NM_002228.4.

### 4.12. Statistical Analysis

A one-way unpaired t-test was used to estimate statistical significance. A *p* value < 0.05 (*) was considered statistically significant.

## 5. Conclusions

We found that the human NANOS1 protein plays an anti-apoptotic role in germ cells by negatively regulating a set of pro-apoptotic genes (*GADD45A*, *GADD45B*, *GADD45G*, *RHOB*). Release of this negative regulation by the p.[(Pro34Thr);(Ser83del)] mutation could account for switching of NANOS1 activity from an anti-apoptotic to a pro-apoptotic state. Such a mechanism may explain the lack of germ cells in infertile patients carrying that mutation.

## Figures and Tables

**Table 1:** List of pro-apoptotic genes selected from RNA-Seq analysis as downregulated by NANOS1. Expressions of these genes were reduced in TCam-2 cells transfected with the wild-type NANOS1 construct in comparison to cells transfected with the empty vector.

HGNC	Base Mean	log2 Fold Change	Adjusted *p* Value	*p* Value	Standard Error
SIAH1	26.98	−0.78	9.31 × 10^−3^	1.11 × 10^−3^	0.24
GADD45B	311.44	−0.65	6.91 × 10^−4^	3.08 × 10^−5^	0.16
GADD45G	334.60	−0.64	4.47 × 10^−3^	3.97 × 10^−4^	0.18
BCL10	32.64	−0.64	3.32 × 10^−2^	6.68 × 10^−3^	0.24
STK17A	392.07	−0.62	7.51 × 10^−4^	3.48 × 10^−5^	0.15
JUN	234.83	−0.62	1.63 × 10^−3^	1.00 × 10^−4^	0.16
GADD45A	1051.99	−0.55	8.22 × 10^−4^	3.93 × 10^−5^	0.13
TP53BP2	894.20	−0.51	4.09 × 10^−4^	1.57 × 10^−5^	0.12
RHOB	516.17	−0.42	3.00 × 10^−2^	5.78 × 10^−3^	0.15
RIPK1	294.70	−0.41	3.69 × 10^−2^	7.70 × 10^−3^	0.15

## Supplementary Materials

Supplementary materials can be found at https://www.mdpi.com/1422-0067/21/8/3009/s1. **Supplementary Table S1**. List of primers used for RT-qPCR.

## Abbreviations

ACTB	actin beta
cDNA	complementary DNA
FPKM	fragments per kilobase of transcript per million mapped reads
GADD45	growth arrest and DNA damage inducible 45
hid	head involution defective
HRP	horseradish peroxidase
mRNA	messenger RNA
MTS	(3-(4,5-dimethylthiazol-2-yl)-5-(3-carboxymethoxyphenyl)-2-(4-sulfophenyl)-2H-tetrazolium)
MUT	mutated
NIM	NOT1 interacting motif
PGCs	primordial germ cells
POI	premature ovarian insufficiency
RHOB	ras homologue family member B
RNA-Seq	RNA sequencing
RT-qPCR	reverse transcription and quantitative polymerase chain reaction
SCOS	Sertoli cell-only syndrome
skl	sickle
TGCT	testicular germ cell tumors
VCL	vinculin
WT	wild-type
ZnF	zinc-finger domain
