# Supplementary material for: Human NANOS1 Represses Apoptosis by Downregulating Pro-Apoptotic Genes in the Male Germ Cell Line
Source: Int J Mol Sci. 2020 Apr 24;21(8):3009. doi: 10.3390/ijms21083009 (PMC7215683; doi:10.3390/ijms21083009)
Supplement: Supplementary file 1 [file ijms-21-03009-s001.pdf]

Supplementary Table S1. List of primers used for RT-qPCR. For primers, F indicates forward and R – revers.

Gene	Primer	Sequence 5′ → 3′	Primer Length [nt]	Amplicon Length [nt]	Annealing Temperature [°C]
BCL10	F	AATACCATCTTCTCTTCA	18	91	56 °C
	R	TTCCTTCTTCTTCTAACT	18		
GADD45A	F	GTGACGAATCCACATTCATCTC	22	90	56 °C
	R	CCATTGATCCATGTAGCGACTT	22		
GADD45B	F	AAGTTGATGAATGTGGAC	18	135	56 °C
	R	GATGTTGATGTCGTTGTC	18		
GADD45G	F	TCAGCCAAAGTCTTGAAC	18	107	56 °C
	R	ATCAGCGTAAAATGGATCT	19		
JUN	F	ACGACCTTCTATGACGAT	18	91	56 °C
	R	TCTGTTTCAGGATCTTGG	18		
RHOB	F	TGCTGATCGTGTTCAGTAAG	20	91	56 °C
	R	TTGCCGTCCACCTCAATG	18		
RIPK1	F	AGAGAAGTCGGATGTGTA	18	98	56 °C
	R	TTATCAACTGCTGCTCAC	18		
STK17A	F	TGAACTAGCACAAGACAATCCT	22	91	56 °C
	R	AGCAGCATATTCCAGAACTAAGA	23		
TP53BP2	F	ACCAGAGCAGTGAAGATA	18	135	56 °C
	R	CTGAAGGTGGCTGATTAG	18		
SIAH1	F	CATCAGCATAAGTCCATTACAAC	23	82	56 °C
	R	CAACAGCACCAGGAAGATTA	20		
ARNT	F	CCACAG-GAACTCTTAGGAA	19	117	56 °C
	R	CATGACAGACAGCACTTG	18		
GAPDH	F	CGGAGTCAACGGATTTGGTCGTAT	24	307	56 °C
	R	AGCCTTCTCCATGGTGGTGAAGAC	24		
RPL13	F	CCTGGAGGAGAAGGAGGGAAAGA GA	25	102	56 °C
	R	TTGAGGACCTCTGTGTATTTGTCAA	25		
UBC	F	ATTTGGTGCGCGGTTCTTG	19	114	56 °C
	R	TGCCTTGACATTCTCGATGGT	21		
